# Effects of 5-Hydroxymethylcytosine Epigenetic Modification on the Stability and Molecular Recognition of VEGF i-Motif and G-Quadruplex Structures

**DOI:** 10.1155/2018/9281286

**Published:** 2018-05-16

**Authors:** Rhianna K. Morgan, Michael M. Molnar, Harshul Batra, Bethany Summerford, Randy M. Wadkins, Tracy A. Brooks

**Affiliations:** ^1^School of Pharmacy, Department of BioMolecular Sciences, Division of Pharmacology, University of Mississippi, University, MS 38677, USA; ^2^Department of Chemistry and Biochemistry, University of Mississippi, University, MS 38677, USA; ^3^School of Pharmacy and Pharmaceutical Sciences, Department of Pharmaceutical Sciences, Binghamton University, Binghamton, NY 13902, USA

## Abstract

Promoters often contain asymmetric G- and C-rich strands, in which the cytosines are prone to epigenetic modification via methylation (5-mC) and 5-hydroxymethylation (5-hmC). These sequences can also form four-stranded G-quadruplex (G4) or i-motif (iM) secondary structures. Although the requisite sequences for epigenetic modulation and iM/G4 formation are similar and can overlap, they are unlikely to coexist. Despite 5-hmC being an oxidization product of 5-mC, the two modified bases cluster at distinct loci. This study focuses on the intersection of G4/iM formation and 5-hmC modification using the vascular endothelial growth factor (VEGF) gene promoter's CpG sites and examines whether incorporation of 5-hmC into iM/G4 structures had any physicochemical effect on formation, stability, or recognition by nucleolin or the cationic porphyrin, TMPyP4. No marked changes were found in the formation or stability of iM and G4 structures; however, changes in recognition by nucleolin or TMPyP4 occurred with 5-hmC modification wherein protein and compound binding to 5-hmC modified G4s was notably reduced. G4/iM structures in the VEGF promoter are promising therapeutic targets for antiangiogenic therapy, and this work contributes to a comprehensive understanding of their governing principles related to potential transcriptional control and targeting.

## 1. Introduction

Guanine- and cytosine-rich DNAs are capable of forming higher order, noncanonical, intramolecular structures known as G-quadruplexes (G4s) and i-motifs (iMs), respectively. iM/G4 forming sequences are common throughout the genome with high abundances in promoters and 5′ untranslated regions (UTRs) of both oncogenes and tumor suppressor genes [1, 2]. These structures are associated with an array of human diseases, including cancer and neurodegeneration [3, 4]. They are also highly polymorphic, both across regions of DNA and within one sequence, and vary in the number of intercalated-cytosines and stacked G-tetrads, as well as in loop length and directionality [5]. It has been suggested that short loop lengths are favorable for G4s, while iMs are stable with long loops, but there are many more factors playing a role in these formations [5]. Noncanonical DNA structures have been characterized as transcriptional regulators, acting as either activators or suppressors depending on the gene involved. Most iMs and G4s identified to date act as transcriptional suppressors by hindering RNA polymerase activity and/or transcription factor binding. Such structures span the hallmarks of cancer; thus, most research focuses on utilizing small iM/G4-stabilizing molecules in order to decrease tumor activity [3–7].

During replication or transcription, the complementary strands of gene promoters are separated. Separation of the double-stranded DNA (dsDNA) helix to single-stranded DNA (ssDNA) has the potential to form iM/G4 structures [8–11]. At physiological pH, both iMs and G4s have previously been described to form with dehydrating and crowding reagents or torsional stress aiding in the stability of structure formation [12–14]. Overall, the topology of iMs and G4s depends on the environment, specifically pH, dehydrating conditions, and epigenetics [13, 15, 16]. Noncanonical DNA structures can form in an equilibrium with B-DNA duplexes, depending on the sequence and surrounding nuclear conditions. As the interest in iM/G4 function in biological systems grows, understanding the structural dynamics in the presence of epigenetic modification will help to clarify what occurs in the cellular milieu.

Modifications, such as methylation or hydroxymethylation, to bases in a DNA sequence are epigenetic factors regulating gene expression. Unique regions in a genome prone to these types of modifications are clustered cytosine-guanine-rich sequences, known as CpG islands. These islands consist of repeats of a cytosine being placed next to a guanine in the same sequence with only a phosphate separating the two nucleotides [17, 18]. The occurrence of CpG islands in mammals is quite low, less than 1% in humans [19]. DNA methyltransferase (DNMT) enzymes, usually with the methyl group transferred from S-adenosylmethionine (SAM), generate the methylated cytosine [20]. The first step of cytosine demethylation is conversion of the methyl (5-mC) to a hydroxymethyl (5-hmC) by the ten-eleven-translocation (TET) proteins. From there, the cytosine is further modified until the base is removed and DNA repair proteins fill the basic site with an unmodified cytosine [21, 22]. When CpG islands occur, modification of a cytosine increases the probability of altering gene expression [23]. Transcriptional activity is positively correlated with 5-hmC density and negatively correlated with 5-mC density [24, 25].

In spite of 5-hmC being a modification of 5-mC, these two epigenetically modified bases have been described as having differential genomic density and to be nonoverlapping. Often, but not always, CpG islands and iM/G4 formation are capable of occurring in the same sequence; however, 5-mC modification is generally not found to be overlapping with iM/G4-forming sequences [20, 26]. A study of the effects of modification on a G4 found within the Bcl-2 promoter demonstrated a stabilization of the noncanonical structure, highlighting the need to study effects on individual structures within promoters where modification can potentially occur [27].

**Figure 1:**
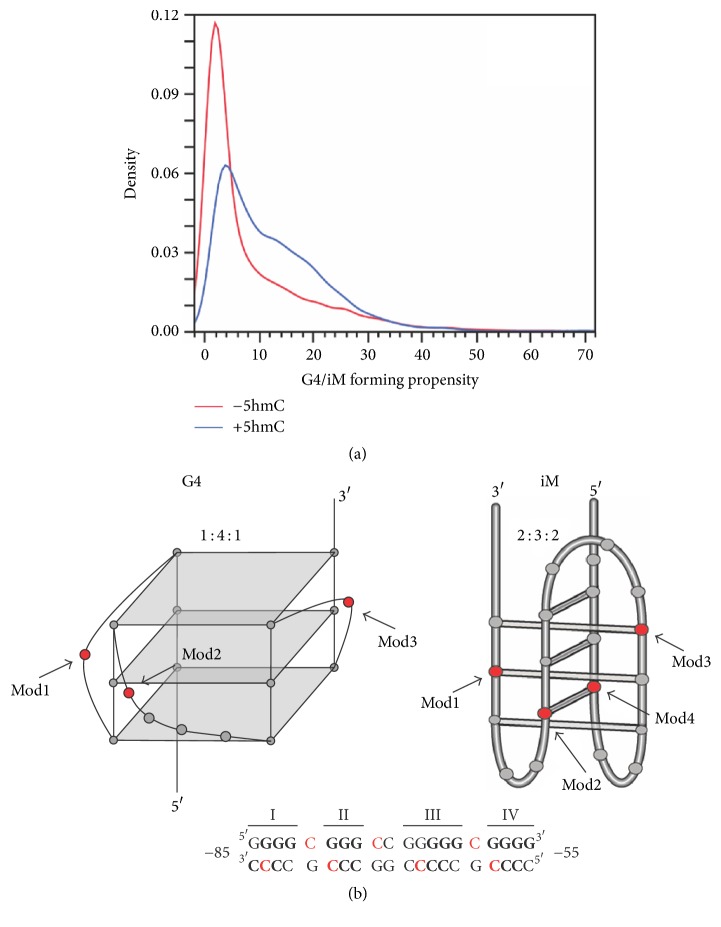


Vascular endothelial growth factor (VEGF) produces a protein that stimulates angiogenesis allowing oxygen restoration to tissue [28]. When overexpressed, VEGF is responsible for the rapid development of new blood vessels to cells, specifically tumor cells. This can cause vascular disease and can help tumors metastasize. VEGF is a key player in tumorigenesis for many cancer cell types making it a promising target for drug therapy. The promoter within this oncogene contains a main cis-element located −85 to −50 bp from the transcriptional start site (TSS). This element is an iM/G4-forming region consisting of five contiguous runs of cytosines/guanines wherein iM and G4 structures form from four contiguous runs, as described in ssDNA and supercoiled plasmids [12, 14, 29–31]; the fifth run has been postulated to have a role in base-excision repair, as the first four runs are resistant [32]. Besides enabling higher order formation, this G/C-rich region close to the TSS contains CpG sequences favorable for cytosine hydroxymethylation. Given the generalized mutual exclusivity of 5-mC and 5-hmC, and also of 5-mC and iM/G4 formation (Figure 1(a)), this study sought to understand the potential effects of 5-hmC modification on the iM and G4 formations of the VEGF promoter. We utilized G- or C-rich G4- or iM-forming oligonucleotides from this sequence containing 5-hmC modifications, as compared to wild-type (iM_WT_ or G4_1-4_), on different relevant cytosines involved in hemiprotonated C:C base-pairing for the iM, or in loop formation within the G4 (Figure 1(b)). Using this model, we sought to elucidate the effects of molecular crowding and dehydration [13, 33, 34], in combination with cationic strength and varying pH, on iM and G4 structure formation, stability, and recognition by proteins and compounds.

## 2. Materials and Methods

### 2.1. Materials

All oligonucleotides were synthesized and purchased from Midland Certified Reagent Co., Inc. (Midland, TX) (Table 1). iM oligonucleotides were stored in 10 mM Tris, 1 mM EDTA buffer at pH 8.0 at −20°C. G4s were stored in autoclaved dH_2_O at 4°C. Acrylamide/bisacrylamide (29 : 1) solution and ammonium persulfate were purchased from Bio-Rad laboratories (Hercules, CA), and N,N,N′,N′-tetramethylethylenediamine was purchased through Fisher Scientific (Pittsburgh, PA). The GelGreen nucleic acid stain was acquired from Phenix Research Products (Candler, NC). All other chemicals were purchased from Sigma-Aldrich (St. Louis, MO).

### 2.2. UV Visual Spectroscopy

The ECD thermal melt profiles of the iM oligonucleotides (2–5 *μ*M) in 30 mM cacodylate buffers at varying pHs (5.4–8.0) with and without various amounts of KCl and acetonitrile (ACN) were validated using absorbance readings at 260 nm collected on Cary 100 UV-Visible Spectrometer (Agilent Technologies, Santa Clara, CA); the pH of each sample was measured in a pH meter immediately before placement in the spectrophotometer. Each solution was first heated to 80°C for 5 minutes and then cooled to 4°C for 2 minutes for two cycles prior to experimental data collection to remove mismatched dimers. The iM thermal denaturation was also monitored by UV-Vis spectroscopy on a Cary 100 UV-Visible Spectrometer (Agilent Technologies, Santa Clara, CA). Recordings were made over the wavelength range 225–350 nm. For thermal denaturation, increasing temperatures of 20–100°C with a ramp rate of 1°C per minute with a 1 minute hold per degree were used [35, 36]. Thermal melt values were determined by nonlinear regression fitting on KaleidaGraph software (Synergy Software, Reading, PA) of the spectral data to determine the midpoint of the transition (*T*_*M*_) and Δ*H* for the folded to unfolded transition.

### 2.3. Electronic Circular Dichroism (ECD)

The G4 oligonucleotides (5 *μ*M) were fixed in 50 mM Tris-HCl (pH 7.4) and iM solutions in 30 mM cacodylate buffers ranging in pH from 5.4 to 8.0, both with varying amounts of KCl and ACN, and 10 *μ*M TMPyP4. Additionally, TMPyP4 effects on G4 stability were examined. Spectra were collected with an Olis DSM-20 spectropolarimeter equipped with a CD 250 Peltier cell holder (Bogart, GA). Recordings were made over the wavelength range 225–350 nm and at increasing temperatures (20–100°C, every 10°C, with a 1 minute hold at temperature before spectra were recorded) in 1 mm quartz cuvette. The ordinate is reported as molar ellipticity (millidegrees); *T*_*M*_s were determined by performing a singular value decomposition (SVD) [37] analysis available with the Olis GlobalWorks software, followed by nonlinear regression fitting to determine *T*_*M*_ using GraphPad Prism software (La Jolla, CA). The iM pK_a_ and cooperativity of the pH driven folding (Hill coefficient) were similarly determined from fitting of the Hill equation [38] using the ECD signal obtained at 298 nm plotted versus pH.

### 2.4. Isolation of Nucleolin Nuclear Extract

HEK-293 cells were plated at a density of 10 × 10^6^ cells per well in a 60 mm dish in 6 mL of Dulbecco complete growth medium (DMEM) with 1x penicillin/streptomycin solution and 10% fetal bovine serum. Cells were transfected with 20 *μ*g of nucleolin expressing plasmid upon reaching approximately 70–80% confluency. After 48 hrs, nuclear extraction was performed using the Thermo Scientific NE-PER Nuclear and Cytoplasmic Extraction kit. Briefly, cells were harvested with trypsin-EDTA and centrifuged at 4,000 rpm. The pellet was resuspended in 1x PBS and transferred to a 1.5 mL microcentrifuge tube before pelleting by centrifugation at 500 ×g for 2-3 mins. Ice-cold CER I was added and vortexed vigorously for 15 sec before being incubated on ice for 10 mins. Again, ice-cold CER II was added and vortexed for 5 seconds before a second incubation on ice for 1 min. Next, the sample was centrifuged for 5 mins at 16,000 ×g. The cytoplasmic extract was discarded and NER was added to the pellet. The sample was vortexed for 15 sec every 10 mins, for a total of 40 mins. Lastly, the sample was centrifuged at 16,000 ×g for 10 mins, and the supernatant (nuclear extract) was collected and stored at −80°C until use.

### 2.5. Electrophoretic Mobility Shift Assay (EMSA)

Non-FAM-labeled oligonucleotides were prepared with 140 mM KCl, 10 mM Tris-HCl (pH 5.4, 6, or 7.4) and 30–40% (v/v) ACN. The solutions were denatured by heating to 95°C for 5 minutes and then rapidly cooled for 5 minutes and repeated for five cycles to induce G4/iM formation. Upon addition of GelGreen nucleic acid stain and nondenaturing loading dye, the samples were loaded on a 10% native polyacrylamide gel. After running at 100 V, the gel was visualized under blue light LED using a FOTO/Analyst® Investigator FX Imager (Hartland, WI). A G4 knockout oligonucleotide was utilized as a linear control for G4 and iM migration. Nucleolin (0–30 *μ*g) protein/G4 binding was examined using the same non-FAM-labeled oligonucleotides (4 *μ*M) prepared in 2x binding buffer (40 mM Tris-HCl, pH 7.5, 40 mM NaCl, 10 mM KCl, 10% (w/v) glycerol, 2 mM EDTA, and 2 mM DTT), 1 *μ*g/*μ*L BSA, and 0.5 *μ*g/*μ*L calf thymus DNA. The samples were incubated on ice for 30 min prior to the addition of oligonucleotides. Following the addition, samples were incubated on ice again for 20 min. Eva green nucleic acid stain and nondenaturing loading dye were added to each sample and ran on a 0.5% agarose gel at 100 V for 40 min at 4°C. The gel was visualized using the same blue light LED of the imager aforementioned.

## 3. Results

### 3.1. 5-hmC Modification Effects on the pK_a_ of the VEGF Promoter iM

Previously, we noted that the inclusion of a single 5-hmC residue in a mutant iM-forming sequence from the c-MYC gene promoter greatly enhanced the cooperativity of folding of the sequence in response to pH [18]. Although the cytosine queried previously was not in a CpG island and thus is unlikely to be modified under normal intracellular conditions, this cooperative folding was found with 5-hmC, but not 5-mC. This observation led to us to expand the 5-hmC work in a more physiologically relevant sequence and to study both the C- and G-rich strand formations. Here, we examined four different CpG positions in the C-rich sequence of DNA from the VEGF promoter that is known to fold into an iM [10]. The ssDNA oligonucleotides containing modifications were identified based on CpG mono- or di-nucleotide motifs. Cytosines modified for iM_mod2_ and iM_mod4_ (and G4_mod2_ described below) were chosen because cytosine modifications occur on the most distal nucleotide in a  CCG_(1-2)_ motif [25].

The DNA's ability to fold into its iM when a 5-hmC modification is present on the oligonucleotide was examined for pH dependence over the range 5.0 to 8.1 using electronic circular dichroism (ECD). The maximum circular dichroism signal was seen at 290 nm and minimum at 260 nm indicating iM formation occurs when 5-hmC modification is present at each CpG site (Figure S1). Using the maximum signal at 290 nm, the change in ellipticity as pH increased was used to determine the pK_a_ and Hill coefficient. The pK_a_ of an iM is generally accepted to be defined as the pH where 50% of the DNA strands in the sample are folded. Each 5-hmC modified and control sequences had a pK_a_ at 6.05 ± 0.06. Also, in contrast to our observations with the iM from c-MYC [20], we did not observe a significant shift in the cooperativity of the pH-induced denaturation; all strands had a fitted Hill coefficient of −1.54 to −2.17, with no discernable trend between Hill coefficient value and 5-hmC location (Table 2).

### 3.2. 5-hmC Modification Effects on Thermal Stability and Equilibrium

Thermal melting (*T*_*M*_) experiments were done at pH 5.4 on modified oligonucleotides in the absence or presence of KCl alone or with the dehydrating agent, acetonitrile (ACN) (Table 3). No notable changes in thermal stability, as defined by *T*_*M*_, occurred with epigenetic modification or cosolvent condition variation. As a general trend, Δ*H* decreased with increasing KCl concentrations by 10–22%, although there were exceptions with modifications at the second and third positions whose Δ*H* increased by up to 32%. The addition of ACN generally increased the Δ*H* by 20–30%, with the exception of a 14% decrease with iM_mod2_ in the presence of 100 mM KCl. From this experiment, it can be concluded that there are no global effects on iM thermal stability induced by either 5-hmC modification or cosolvent condition; few notable changes were noted in folding dynamics, particularly with 5-hmC incorporation into positions two or three in the presence of KCl.

Similar thermal melting studies of iMs were done at pH 6.1 (the pK_a_). The ensemble of wild-type and modified iM oligonucleotides require less thermal energy to unfold (Table 4) compared to pH 5.4, since half of the ensemble was unfolded due to pH. At pH 6.1, there is also a general trend of lower thermal stability in conditions containing KCl across all oligonucleotides. The addition of KCl also decreased Δ*H* of iM_mod1_, iM_mod2_, and iM_mod4_ by more than 50%. Moreover, the combination of KCl and ACN in iM samples decreased both *T*_*M*_ and Δ*H*, as compared to either milieu change alone or versus control (Table 4 and Figures S2–S11).

### 3.3. 5-hmC Modification Effects on G4 Formation and Stability

Overall G4 formation and stability, as affected by 5-hmC modifications, was examined by ECD. The wild-type form, G4_1-4_, forms a prominent parallel structure as evidenced by the positive cotton effect at 265 nm (Figure S12(A)) [12, 30, 31, 39]. G4 formation was induced in the presence of cations and osmolytes, using 100 mM KCl and 40% ACN to induce very stable structures as a measure of maximal formation (Table 5). G4_1-4_ thermal stability increased from 62 to 84°C in 100 mM KCl and exceeded 100°C with the addition of 40% ACN. Similarly, G4_mod1_ increased from 50 to 82°C with 100 mM KCl, and G4_mod3_ increased from 73 to 84°C. Both of these latter modifications have increased thermal stability in the presence of 40% ACN to 95°C or more. G4_mod2_, however, does not form a strong structure in the absence of any cosolvents; a parallel orientation is only apparent when KCl and ACN are added (Figure S12(A)). In the presence of 100 mM KCl, G4_mod2_ has a melting temperature of 76°C, which increased to greater than 100°C with 40% ACN. A knockout oligonucleotide, G4_MT_, with G-to-T mutations in each guanine run within the wild-type sequence was used as a negative control; disruption of G4 formation was confirmed by ECD in the presence of 100 mM KCl with and without 40% ACN (Figure S12(B)).

Further studies were performed to examine G4 formation in the presence of cosolvents to mimic more physiologically relevant conditions, such as dehydration and structures existing in an equilibrium with unfolded DNA. Appropriate cosolvent concentrations were determined by evaluating the minimal concentration necessary (0–40% (v/v) ACN) to induce marked G4 formation, as determined by ECD spectra of G4_1-4_ (Figure S13). The concentrations of 10 mM KCl and 30% ACN were chosen to investigate modified G4 formation as moderately stabile conditions (analogous to pK_a_ for iM structures). We monitored the effect of these cosolvents on G4 stability with epigenetic modifications present compared to wild-type (Figure S14). G4_1-4_ thermal stability increased from 67 to 83°C upon addition of 30% ACN. Similar to wild-type, the presence of KCl and/or ACN increased the stability of G4s containing 5-hmC modifications as well (Table 6). G4_mod1_ was enhanced from 68°C with 10 mM KCl to 80°C with 30% ACN. Melting temperatures of G4_mod2_ increased from 68 to 84°C upon ACN addition, as compared to KCl control. G4_mod3_, like G4_mod1_, increased stability from 67 to 80°C with ACN. Cumulatively, a change in G4 stability in the presence of an epigenetic modification was only noted with G4_mod2_ in 100 mM KCl (*T*_*M*_ of 76°C) as compared to wild-type G4_1-4_ (*T*_*M*_ of 84°C). Overall, 5-hmC modification did not markedly affect G4 formation or stability.

### 3.4. Inter- and Intramolecular Higher Order DNA Formations

**Figure 2:**
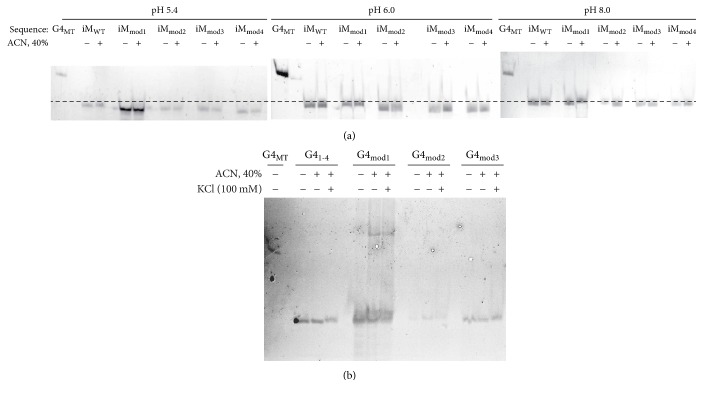


Electrophoretic mobility shift assays (EMSA) were then used to determine inter- versus intramolecular iM and G4 structure formation (Figure 2). The knockout oligonucleotide was used as a linear reference to align the iM EMSAs and to indicate G4 migration patterns. Each iM sample was examined at distinct pHs in order to determine the dependence of C-rich structure formation on molecular interactions with surrounding protons. Each G4 sample was examined in the absence and presence of monovalent cation to stabilize the structure. Both iM and G4 structures were also examined in the presence of dehydrating cosolvent (40% ACN).

Generally, iM species at pH 5.4 migrated faster than the same sequences at pH 8.0, where they have been determined to be linear ssDNA by ECD (Figure S1). All species at pH 6.0 (the pK_a_) span the distance between the pH 5.4 and the pH 8.0 bands, in agreement with a mixed structured and unstructured population. None of the 5-hmC modifications markedly changed migration patterns, nor did the addition of 40% ACN (Figure 2(a)).

G4_1-4_ and the 5-hmC-modified G4s migrated further than the knockout reference, MT, suggesting the existence of intramolecularly folded species. Further migration is seen when 100 mM KCl is added; however, the shift is not marked. When 40% ACN is added, slower migration of each sequence displays banding above the corresponding control and 100 mM KCl samples. Notably, G4_mod1_, with 100 mM KCl alone and in the presence of 40% CAN, demonstrates the formation of intermolecular structures, which may be correlated to the change in enthalpy noted with G4_mod1_ (Table 6). This decrease in enthalpy likely signifies an increase in the number of isoforms existing, which may include a cohort of inter- and intramolecular G4 species. No other sequence mediated the formation of intermolecular species; G4_mod2_ and G4_mod3_ closely resembled wild-type G4_1-4_ (Figure 2(b)). Collectively, 5-hmC modifications on cytosines involved in iM hemiprotonated C:C bonds or in loop formation of G4s do not impair the stability or formation of these DNA structures.

### 3.5. 5-hmC Modification Modulates G4 Recognition

**Figure 3:**
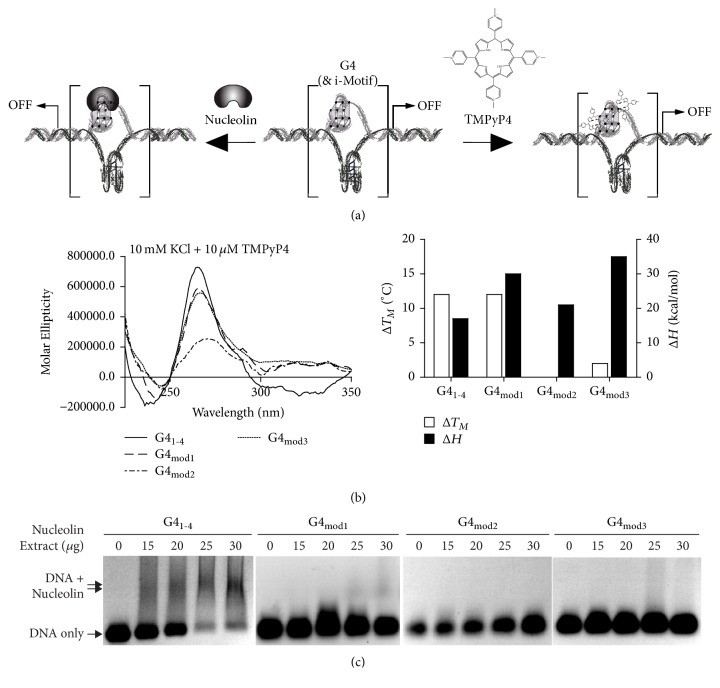


We further examined whether epigenetic modifications impact structure recognition by G4-stabilizing small molecules and regulatory binding proteins. The universal G4-stabilizing cationic porphyrin, TMPyP4, was used to monitor compound recognition (Figure 3(a)). Previous data demonstrated stabilization of the VEGF G4_1-4_ by TMPyP4, with a related decrease in transcription [27, 28]. TMPyP4 (10 *μ*M) was incubated with the VEGF G4 with and without 5-hmC modifications; the compound increased both G4_1-4_ and G4_mod1_ stability by 12°C; in comparison, neither G4_mod2_ nor G4_mod3_ were stabilized by the porphyrin. No changes in Δ*H* were observed across the WT or modified sequences (Figure 3(b)).

To investigate the effect of 5-hmC modification on protein recognition, the binding of nucleolin was examined. Nucleolin has been reported to bind the VEGF promoter in both negative supercoiled plasmids and chromosomal DNA [14]. Nuclear extract from HEK-293 cells transfected with nucleolin was incubated with G4s. EMSA was performed to visualize protein: DNA binding. Nucleolin demonstrated concentration-dependent binding to the wild-type G4 structure, with 75% of DNA bound in the presence of 30 *μ*g of nucleolin extract. Decreased binding to G4_mod1_ (~30%) was seen at the higher concentrations of nucleolin (25 and 30 *μ*g); however, neither G4_mod2_ nor G4_mod3_ were bound by nucleolin at concentrations of extract up to 30 *μ*g (Figure 3(c)). Collectively, this suggests that epigenetic modifications can influence molecular recognition by both small molecules, as seen with TMPyP4, or by regulatory binding proteins, as observed with nucleolin. The propensity of these regions to exist in transient states of epigenetic modification, and its effects on higher order DNA formation and recognition, is a field that requires more study. From the presented data; however, it is clear that G4s, and likely iMs, with CpG islands demonstrate alternative recognition patterns that ought to be taken into consideration in drug discovery research and in mechanistic studies of developing compounds in vitro and in vivo.

## 4. Discussion

G/C-rich DNA is capable of forming higher order DNA structures and is often a template for epigenetic modification. It has been shown that methylated cytosines are generally nonoverlapping with G4-forming regions [26], but when they coincide, there is a stabilization of the G4 [27]. In addition, methylated cytosines and hydroxymethylated cytosines, despite being modifications of each other, are found in mutually exclusive regions of the genome and differentially correlate with transcriptional activity. Given the exclusivity of 5-mC with both iM/G4-forming regions and with 5-hmC, the overall purpose of this study was to examine the effects of 5-hmC modification on the formation, stability, and recognition of secondary DNA structures. As a physiologically relevant model, we focused on the structures in the promoter of the VEGF gene, which have been well-described for both iM and G4 formation [12, 14, 29–31] and containing natural CpG islands [40]. Cytosine modification of this promoter has been shown to vary with pathophysiological states [41, 42]. Moreover, transcription is negatively regulated by methylation and positively regulated by hydroxymethylation, which have specifically been shown to be mutually exclusive in the VEGF promoter [41, 42].

Overall, 5-hmC modifications did not markedly affect iM or G4 formation or stability. The most notable structural affect was the identification of intermolecular G4 formation with modification of the most 5′ CpG island. The recognition of the G4 by a stabilizing compound, TMPyP4 [30, 31], and protein, nucleolin [14], was also examined. Remarkably, with the exception of the intermolecular forming G4_mod1_, TMPyP4 stabilization was abolished by 5-hmC modification, as was nucleolin recognition; protein recognition of G4_mod1_ was markedly reduced. Similar studies were not performed with the VEGF iM, as stabilizing proteins and selective compounds are not fully established, as they are for the G4. Our studies suggest that even in the absence of marked structural iM/G4 changes with epigenetic modification, overall biological effects related to secondary structure regulation of transcription may still occur due to a change in endogenous and exogenous recognition.

Although 5-mC and G4 formation have been shown to be nonconcurrent [26], a study was undertaken to examine the effect of methylation on G4 structure, formation, and function in the Bcl-2 promoter [27]. This region also contains naturally occurring CpGs, capable of being methylated. In that study, methylation of all four internal cytosines that are part of CpGs altered the G4 structure, converting it from a mixed species to a parallel formation, and increasing its thermal stability by 9°C. Previous works on a nonbiologically occurring intermolecular G4 demonstrated that trimethylation of loop cytosines formed a more stable structure than the unmethylated version [38]. iM studies with a modified sequence from the MYC promoter, which does not contain any native CpG sequences, demonstrated slight enhancement in thermal stability and pK_a_ with a single 5-mC modified position, and slightly decreased stability (both thermal and pH-dependent) with a 5-hmC modification at the same location, both in the absence of cosolvents. In the presence of varying cosolvents, any effects on thermal stability mediated by epigenetic changes were lost [20]. It is important to note that G4-forming regions within promoters are more likely to contain 5-hmC than 5-mC modifications (Figure 1(a)) [20, 26]. In the study presented here, we focused only on the 5-hmC modification and examined the effect on both the C-rich (iM-forming) and G-rich (G4-forming) sequences with naturally occurring CpGs. Modifying one cytosine at a time, we monitored pH-dependency, thermal stability, and the effect of cosolvents. Overall, our findings demonstrate a lack of marked changes in G4 or iM formation. The effect of cytosine modification on iM/G4 formation is largely understudied. Effects may be sequence-specific, with variations in each promoter sequence; structure specific, with varying effects on intermolecular, mixed, or antiparallel-predominant structures; or epigenetic modification specific, varying with methylation and hydroxymethylation. To our knowledge, the present study is the first to examine the effects of epigenetic modification on compound and protein recognition, and more work is needed to be established if the lack of G4 recognition with 5-hmC modifications in the loop region is a sequence-specific effect, an epigenetic modification-specific effect, or a consistent phenomenon.

## 5. Conclusion

Previous literature described epigenetic modification of CpG islands in induced pluripotent stem cells and endothelial stem cells and their correlation to gene expression [25]. Endothelial stem cells play key roles in vasculogenesis and angiogenesis, as they form the lining of blood vessels. VEGF is overexpressed by tumors, secreted into the local milieu, and acts upon nearby endothelial cells to trigger angiogenesis in order to increase oxygen levels and provide essential nutrients, mediating tumor growth, invasion, and metastasis [43–45]. Pathological states, such as intracerebral hemorrhage and preterm preeclampsia, have been shown to modulate VEGF expression through epigenetic modulation of the promoter [41, 42]. In particular, methylation of the VEGF promoter has been shown to correlate with a decrease in transcription, and hydroxymethylation coincides with an increase in gene expression [42]. Here, we investigated the iM/G4-forming region within the cis-element on the VEGF promoter which has previously been identified as an antiangiogenic target for therapeutic development [14, 30, 39, 46, 47]. The iM forms a tri-intercalated structure with loops lengths of 2 : 3 : 2 and the G4 forms a tristacked structure with parallel loop configurations of lengths 1 : 4 : 1; stabilization of secondary DNA structures in the promoter has been shown to silence transcription [12, 31]. The iM- and G4-forming sequences contain several CpG sites, so for drug discovery purposes, it is necessary to explore physiological factors, like epigenetic modifications, that can occur in CpG islands that may affect iM/G4 formation and stability [40]. The current study examined the effects of 5-hmC modifications on the VEGF iM/G4-forming region within the promoter and learned that such modifications do not impact overall G4/iM formation. However, these modifications do alter G4 structure recognition by a pan-stabilizing compound, TMPyP4, and the regulatory binding protein, nucleolin. This suggests that drug discovery efforts should consider the presence of epigenetic modifications on the G4, and likely the iM, of interest to consider all potential biological interactions. These studies add to the understanding of the mechanism behind gene regulation via epigenetic modifications.

## Figures and Tables

**Table 1:** The promoter sequence (24 mer) of the VEGF gene and its modifications for both G4/iM-forming regions. **X** denotes the 5-hydroxymethylated cytosine of each oligonucleotide sequence.

iM_WT_	5′GACCCCGCCCCCGGCCCGCCCCGG3′
iM_mod1_	5′GACCC**X**GCCCCCGGCCCGCCCCGG3′
iM_mod2_	5′GACCCCGCCC**X**CGGCCCGCCCCGG3′
iM_mod3_	5′GACCCCGCCCCCGGCC**X**GCCCCGG3′
iM_mod4_	5′GACCCCGCCCCCGGCCCGCC**X**CGG3′

G4_MT_	5′CCGTTGCGTGCCGTTTGCGTTGTC3′
G4_1-4_	5′CCGGGGCGGGCCGGGGGCGGGGTC3′
G4_mod1_	5′CCGGGG**X**GGGCCGGGGGCGGGGTC3′
G4_mod2_	5′CCGGGGCGGG**X**CGGGGGCGGGGTC3′
G4_mod3_	5′CCGGGGCGGGCCGGGGG**X**GGGGTC3′

**Table 2:** Change in molar ellipticity (290 nm) of the electronic circular dichroism (ECD) spectra over the pH range of 5.0 to 8.1. ECD data was used to determine pK_a_ using singular value decomposition (SVD) analysis of pH-dependent folding curves for each 5-hmC modified sample and compared to wild-type control. The Hill coefficient is the fitting parameter that characterizes the cooperativity of folding.

	iM_WT_	iM_mod1_	iM_mod2_	iM_mod3_	iM_mod4_
pKa	6.10 ± 0.04	6.00 ± 0.15	6.10 ± 0.11	6.10 ± 0.05	6.10 ± 0.04
Hill Coefficient	−2.06	−1.60	−1.54	−1.91	−2.17

**Table 3:** Comparison of *T*_*M*_ and Δ*H*° of transition of iM containing 5-hmC ± KCl (100 mM) and ±40% acetonitrile (ACN) at pH 5.4. The unmodified control iM VEGF sequence (iM_WT_) is also shown for comparison. Error measurements are omitted for clarity. Errors in Δ*H*° were less than 12% while errors in *T*_*m*_ values were less than 3%.

	iM_WT_		iM_mod1_		iM_mod2_		iM_mod3_		iM_mod4_	
	Δ*T*_*M*_ (°C)	Δ*H*° (kcal/mol)	Δ*T*_*M*_ (°C)	Δ*H*° (kcal/mol)	Δ*T*_*M*_ (°C)	Δ*H*° (kcal/mol)	Δ*T*_*M*_ (°C)	Δ*H*° (kcal/mol)	Δ*T*_*M*_ (°C)	Δ*H*° (kcal/mol)
Control	61	34	62	34	62	31	61	32	62	33
100 mM KCl Alone	62	27	61	29	61	41	61	35	61	28
40% ACN Alone	63	45	63	41	65	40	63	40	65	39
100 mM KCl + 40% ACN	59	36	62	32	62	35	57	44	62	35

**Table 4:** Comparison of *T*_*M*_ and Δ*H*° of transition of iM containing 5-hmC ± KCl (100 mM) and ±40% acetonitrile (ACN) at pH 6.1. The unmodified control iM VEGF sequence (iM_WT_) is also shown for comparison. Error bars are omitted for clarity and are <7% or 50% of the values shown for *T*_*M*_ and Δ*H*°, respectively.

	iM_WT_		iM_mod1_		iM_mod2_		iM_mod3_		iM_mod4_	
	Δ*T*_*M*_ (°C)	Δ*H*° (kcal/mol)	Δ*T*_*M*_ (°C)	Δ*H*° (kcal/mol)	Δ*T*_*M*_ (°C)	Δ*H*° (kcal/mol)	Δ*T*_*M*_ (°C)	Δ*H*° (kcal/mol)	Δ*T*_*M*_ (°C)	Δ*H*° (kcal/mol)
Control	52	16	52	17	53	15	47	12	51	16
100 mM KCl Alone	48	18	49	8	48	7	44	13	49	7
40% ACN Alone	50	20	48	17	50	18	46	12	49	16
100 mM KCl + 40% ACN	40	10	40	9	41	10	40	5	42	12

**Table 5:** Thermodynamic properties of VEGF G4 modification sequences in the presence and absence of 100 mM KCl and 40% ACN alone or combined. Marked increases in thermal stability were noted upon addition of 100 mM KCl alone and with 40% ACN as compared to their own controls. Thermal stability was not substantially different between 5-hmC modifications and wild-type. No pronounced changes in transition state dynamics were observed. Error bars are omitted for clarity and are <10% of the values shown; ND = not determinable.

	G4_1-4_		G4_mod1_		G4_mod2_		G4_mod3_	
	Δ*T*_*M*_ (°C)	Δ*H*° (kcal/mol)	Δ*T*_*M*_ (°C)	Δ*H*° (kcal/mol)	Δ*T*_*M*_ (°C)	Δ*H*° (kcal/mol)	Δ*T*_*M*_ (°C)	Δ*H*° (kcal/mol)
Control	62	21	50	12	ND	ND	73	16
100 mM KCl	84	22	82	27	76	30	84	15
100 mM KCl + 40% ACN	>100	25	>100	19	>100	20	95	12

**Table 6:** Thermal stability of VEGF G4 modification sequences at lower concentrations of cosolvents. 30% ACN increased thermal profiles of each modification as compared to their respective controls in 10 mM KCl. No marked changes in enthalpy were observed. Error bars are omitted for clarity and are <10% of the values shown; ND = not determinable.

	G4_1-4_		G4_mod1_		G4_mod2_		G4_mod3_	
	Δ*T*_*M*_ (°C)	Δ*H*° (kcal/mol)	Δ*T*_*M*_ (°C)	Δ*H*° (kcal/mol)	Δ*T*_*M*_ (°C)	Δ*H*° (kcal/mol)	Δ*T*_*M*_ (°C)	Δ*H*° (kcal/mol)
Control	62	21	50	12	ND	ND	73	16
10 mM KCl	67	29	68	29	68	27	67	25
10 mM KCl + 30% ACN	83	33	80	24	84	24	80	20

## Data Availability

Either all data referred to in our manuscript are presented or the references are implicated with the data available in the published figures/paper.

## Supplementary Materials

Supplementary Materials Figure S1: pH dependence of iM formation in the native (iM_WT_) and modified (iM_mod1–4_) sequences. ECD spectra of all C-rich sequences were obtained at a range of pHs (5.0–8.1) at 20°C. Figure S2: ECD spectra of VEGF iM_WT_ at pH 5.4 exposed to 0 KCl (mM)/0% acetonitrile, 100 mM KCl by itself, 40% acetonitrile by itself, and both 100 mM KCl/40% acetonitrile present. Each spectrum was collected at 20°C, the determined *T*_*M*_, and 90°C. Figure S3: ECD spectra of VEGF iM_mod1_ at pH 5.4 exposed to 0 KCl (mM)/0% acetonitrile, 100 mM KCl by itself, 40% acetonitrile by itself, and both 100 mM KCl/40% acetonitrile present. Each spectrum was collected at 20°C, the determined *T*_*M*_, and 90°C. Figure S4: ECD spectra of VEGF iM_mod2_ at pH 5.4 exposed to 0 KCl (mM)/0% acetonitrile, 100 mM KCl by itself, 40% acetonitrile by itself, and both 100 mM KCl/40% acetonitrile present. Each spectrum was collected at 20°C, the determined *T*_*M*_, and 90°C. Figure S5: ECD spectra of VEGF iM_mod3_ at pH 5.4 exposed to 0 KCl (mM)/0% acetonitrile, 100 mM KCl by itself, 40% acetonitrile by itself, and both 100 mM KCl/40% acetonitrile present. Each spectrum was collected at 20°C, the determined *T*_*M*_, and 90°C. Figure S6: ECD spectra of VEGF iM_mod4_ at pH 5.4 exposed to 0 KCl (mM)/0% acetonitrile, 100 mM KCl by itself, 40% acetonitrile by itself, and both 100 mM KCl/40% acetonitrile present. Each spectrum was collected at 20°C, the determined *T*_*M*_, and 90°C. Figure S7: ECD spectra of VEGF iM_WT_ at the pKa exposed to 0 KCl (mM)/0% acetonitrile, 100 mM KCl by itself, 40% acetonitrile by itself, and both 100 mM KCl/40% acetonitrile present. Each spectrum was collected at 20°C, the determined *T*_*M*_, and 90°C. Figure S8: ECD spectra of VEGF iM_mod1_ at the pka exposed to 0 KCl (mM)/0% acetonitrile, 100 mM KCl by itself, 40% acetonitrile by itself, and both 100 mM KCl/40% acetonitrile present. Each spectrum was collected at 20°C, the determined *T*_*M*_, and 90°C. Figure S9: ECD spectra of VEGF iM_mod2_ at the pKa exposed to 0 KCl (mM)/0% acetonitrile, 100 mM KCl by itself, 40% acetonitrile by itself, and both 100 mM KCl/40% acetonitrile present. Each spectrum was collected at 20°C, the determined Tm, and 90°C. Figure S10: ECD spectra of VEGF iM_mod3_ at the pKa exposed to 0 KCl (mM)/0% acetonitrile, 100 mM KCl by itself, 40% acetonitrile by itself, and both 100 mM KCl/40% acetonitrile present. Each spectrum was collected at 20°C, the determined *T*_*M*_, and 90°C. Figure S11: ECD spectra of VEGF iM_mod4_ at the pKa exposed to 0 KCl (mM)/0% acetonitrile, 100 mM KCl by itself, 40% acetonitrile by itself, and both 100 mM KCl/40% acetonitrile present. Each spectrum was collected at 20°C, the determined *T*_*M*_, and 90°C. Figure S12: 5-hmC modifications within VEGF G4-forming region allows stable G4 formation with cosolvents. Upon addition of 100 mM KCl alone and combined with 40% ACN, maximal G4 formation occurred compared to control. Also, thermal stability markedly increased with each addition for all sequences. (A) Total G-to-T mutations prevent G4 formation alone and in the presence of 100 mM KCl and 40% ACN (B). Figure S13: determination of cosolvent concentrations needed for maximum VEGF_1–4_ formation. 0–100 mM gradient of KCl was added to wild-type VEGF G4 to determine 10 mM KCl to be the minimal concentration affecting G4 formation markedly. Similarly, 0–40% of ACN, in 10% increments, was added to wild-type resulting in 30% ACN being identified for further study. Figure S14: low concentrations of cosolvents increase thermodynamic stability of modified VEGF structures. 30% ACN increase thermal profiles of each modification as compared to their own control (10 mM KCl).
