# Supplementary material for: Effects of 5-Hydroxymethylcytosine Epigenetic Modification on the Stability and Molecular Recognition of VEGF i-Motif and G-Quadruplex Structures
Source: J Nucleic Acids. 2018 May 16;2018:9281286. doi: 10.1155/2018/9281286 (PMC5976936; doi:10.1155/2018/9281286)
Supplement: Supplementary Materials — Figure S1: pH dependence of iM formation in the native (iMWT) and modified (iMmod1–4) sequences. ECD spectra of all C-rich sequences were obtained at a range of pHs (5.0–8.1) at 20°C. Figure S2: ECD spectra of VEGF iMWT at pH 5.4 exposed to 0 KCl (mM)/0% acetonitrile, 100 mM KCl by itself, 40% acetonitrile by itself, and both 100 mM KCl/40% acetonitrile present. Each spectrum was collected at 20°C, the determined TM, and 90°C. Figure S3: ECD spectra of VEGF iMmod1 at pH 5.4 exposed to 0 KCl (mM)/0% acetonitrile, 100 mM KCl by itself, 40% acetonitrile by itself, and both 100 mM KCl/40% acetonitrile present. Each spectrum was collected at 20°C, the determined TM, and 90°C. Figure S4: ECD spectra of VEGF iMmod2 at pH 5.4 exposed to 0 KCl (mM)/0% acetonitrile, 100 mM KCl by itself, 40% acetonitrile by itself, and both 100 mM KCl/40% acetonitrile present. Each spectrum was collected at 20°C, the determined TM, and 90°C. Figure S5: ECD spectra of VEGF iMmod3 at pH 5.4 exposed to 0 KCl (mM)/0% acetonitrile, 100 mM KCl by itself, 40% acetonitrile by itself, and both 100 mM KCl/40% acetonitrile present. Each spectrum was collected at 20°C, the determined TM, and 90°C. Figure S6: ECD spectra of VEGF iMmod4 at pH 5.4 exposed to 0 KCl (mM)/0% acetonitrile, 100 mM KCl by itself, 40% acetonitrile by itself, and both 100 mM KCl/40% acetonitrile present. Each spectrum was collected at 20°C, the determined TM, and 90°C. Figure S7: ECD spectra of VEGF iMWT at the pKa exposed to 0 KCl (mM)/0% acetonitrile, 100 mM KCl by itself, 40% acetonitrile by itself, and both 100 mM KCl/40% acetonitrile present. Each spectrum was collected at 20°C, the determined TM, and 90°C. Figure S8: ECD spectra of VEGF iMmod1 at the pka exposed to 0 KCl (mM)/0% acetonitrile, 100 mM KCl by itself, 40% acetonitrile by itself, and both 100 mM KCl/40% acetonitrile present. Each spectrum was collected at 20°C, the determined TM, and 90°C. Figure S9: ECD spectra of VEGF iMmod2 at the pKa exposed to 0 KCl (mM)/0% a [file 9281286.f1.pdf]

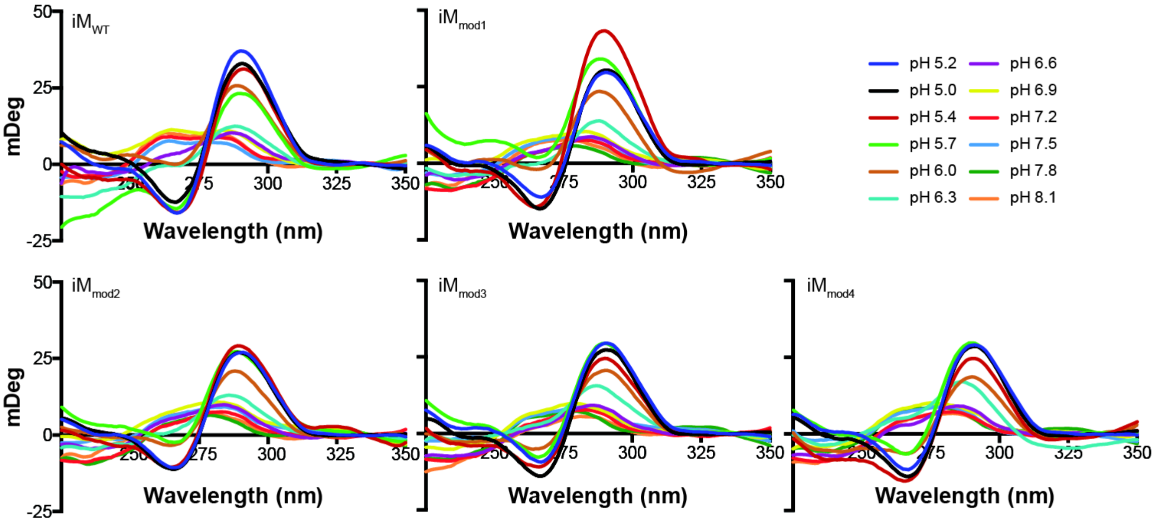

Figure S1. pH dependence of iM formation in the native (iM_{WT}) and modified (iM_{mod1-4}) sequences. ECD spectra of all C-rich sequences were obtained at a range of pH's (5.0 – 8.1) at 20 °C.

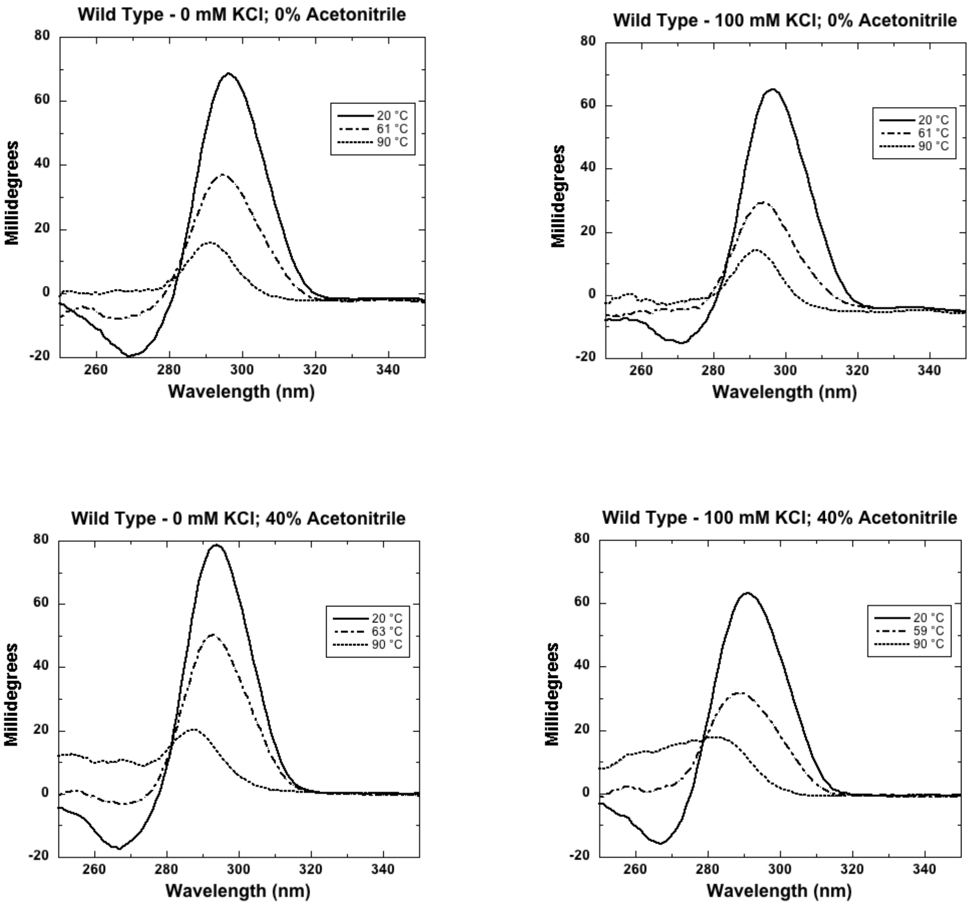

Figure S2. ECD spectra of VEGF iM_{WT} at pH 5.4 exposed to 0 KCl (mM) / 0% acetonitrile, 100 mM KCl by itself, 40% acetonitrile by itself, and both 100 mM KCl / 40% acetonitrile present. Each spectra was collected at 20 °C, the determined T_M, and 90 °C.

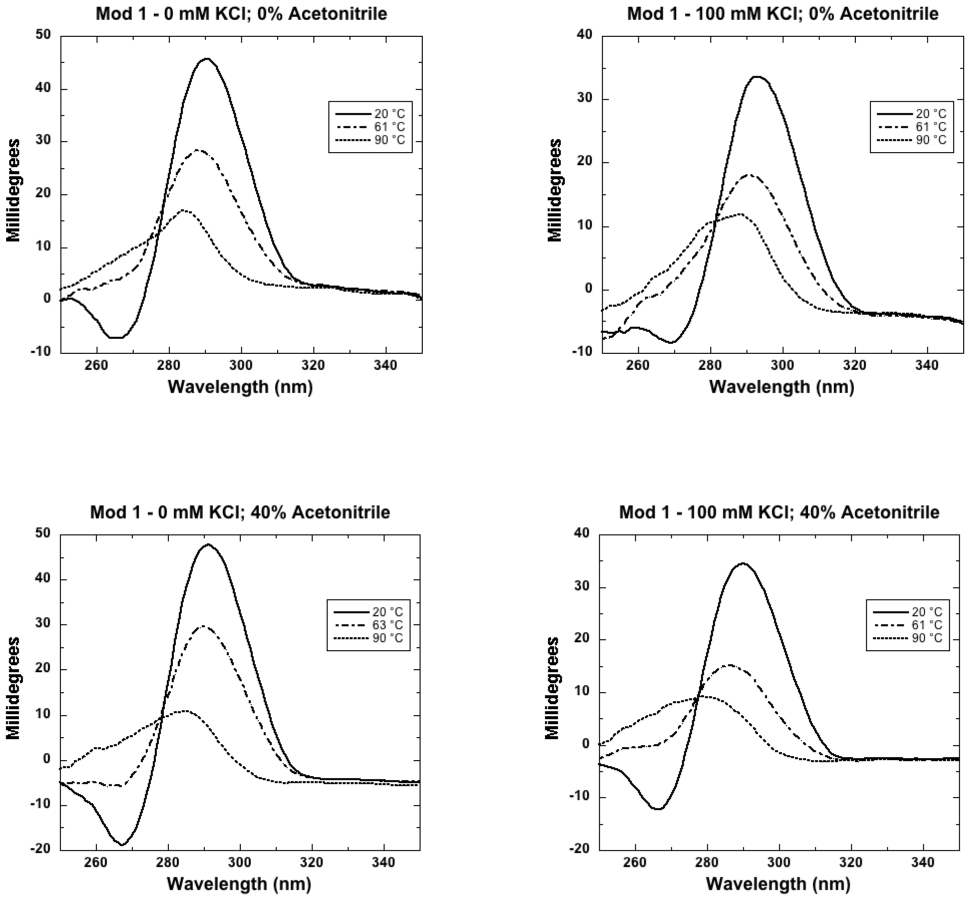

Figure S3. ECD spectra of VEGF iM_{mod1} at pH 5.4 exposed to 0 KCl (mM) / 0% acetonitrile, 100 mM KCl by itself, 40% acetonitrile by itself, and both 100 mM KCl / 40% acetonitrile present. Each spectra was collected at 20 °C, the determined T_M, and 90 °C.

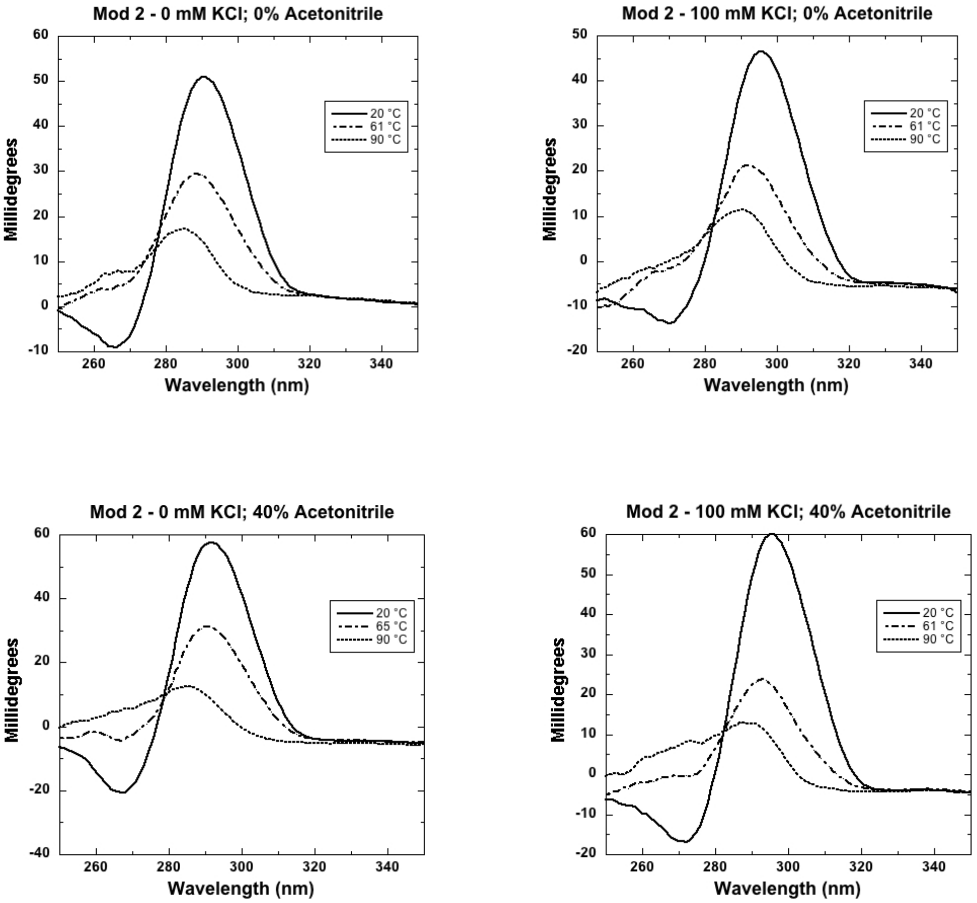

Figure S4. ECD spectra of VEGF iM_{mod2} at pH 5.4 exposed to 0 KCl (mM) / 0% acetonitrile, 100 mM KCl by itself, 40% acetonitrile by itself, and both 100 mM KCl / 40% acetonitrile present. Each spectra was collected at 20 °C, the determined T_M, and 90 °C.

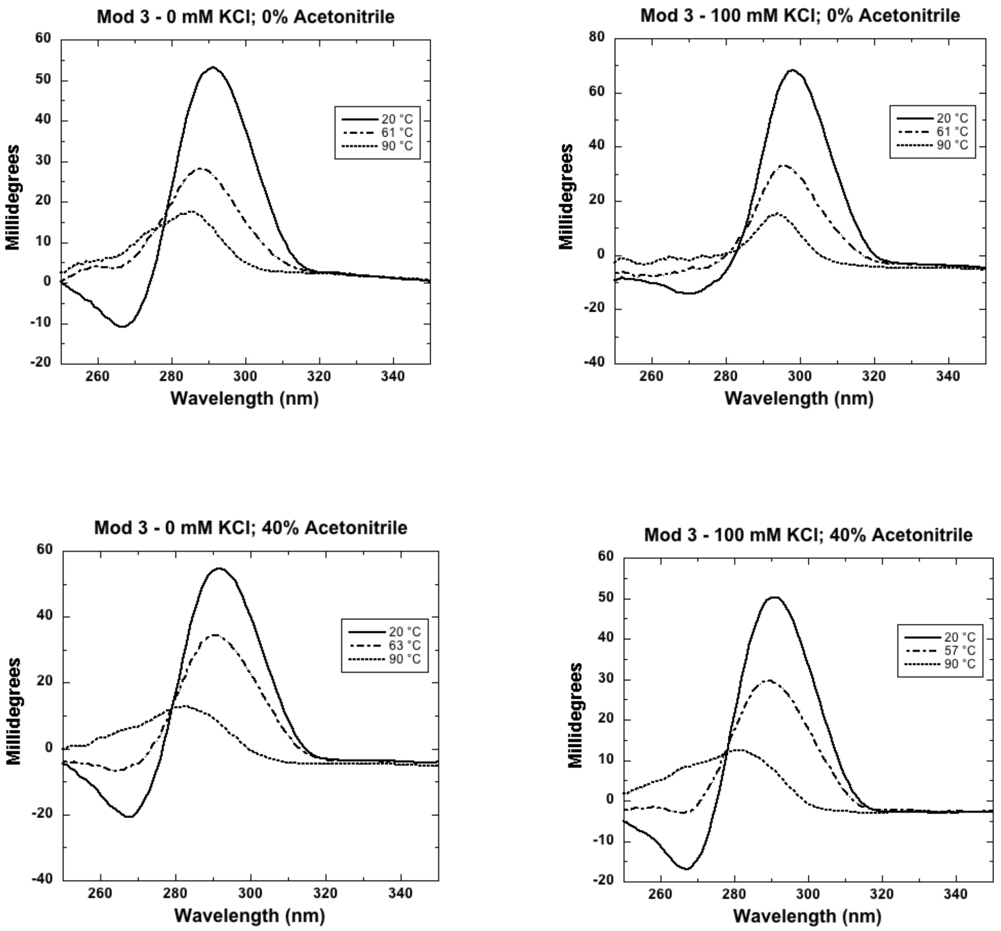

Figure S5. ECD spectra of VEGF iM_{mod3} at pH 5.4 exposed to 0 KCl (mM) / 0% acetonitrile, 100 mM KCl by itself, 40% acetonitrile by itself, and both 100 mM KCl / 40% acetonitrile present. Each spectra was collected at 20 °C, the determined T_M, and 90 °C.

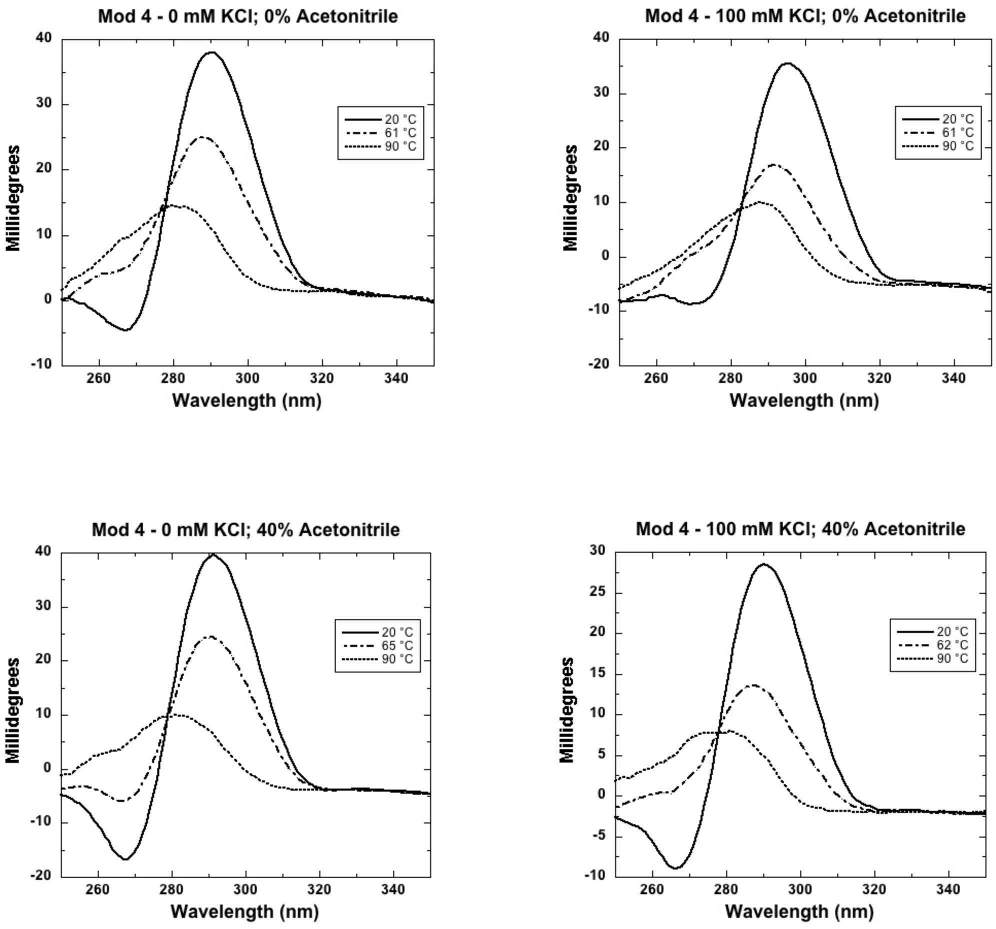

Figure S6. ECD spectra of VEGF iM_{mod4} at pH 5.4 exposed to 0 KCl (mM) / 0% acetonitrile, 100 mM KCl by itself, 40% acetonitrile by itself, and both 100 mM KCl / 40% acetonitrile present. Each spectra was collected at 20 °C, the determined T_M, and 90 °C.

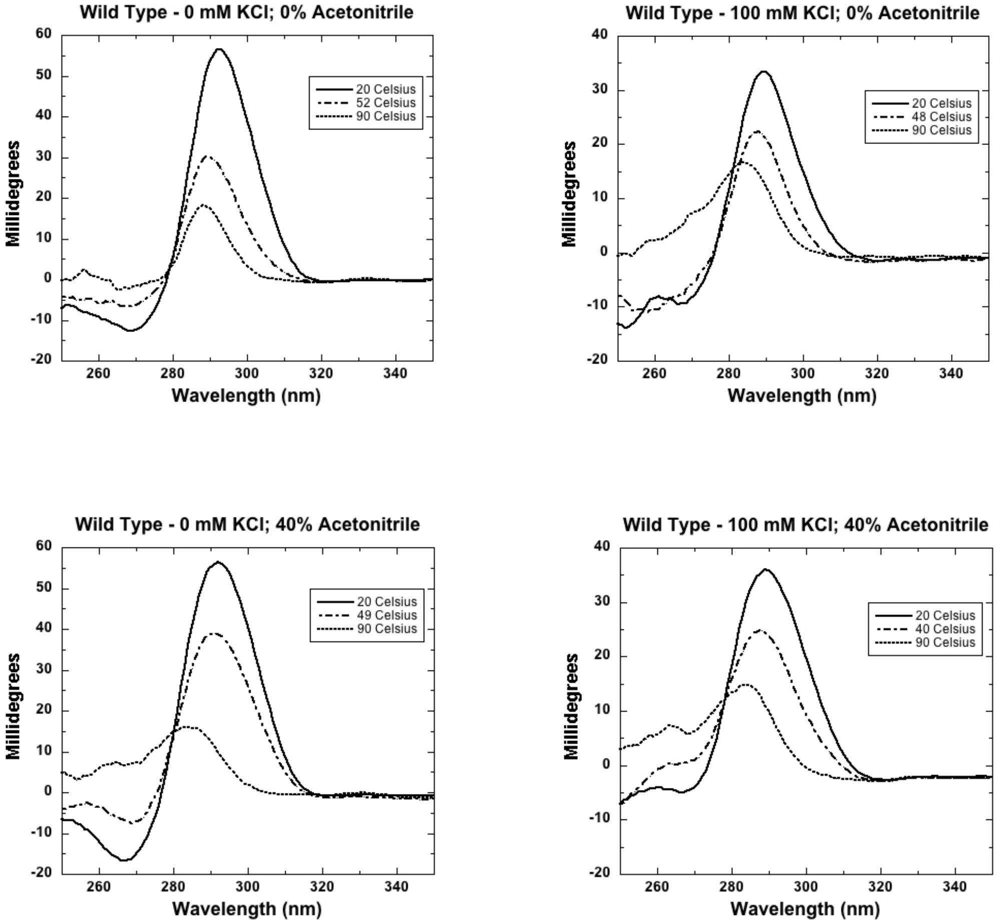

Figure S7. ECD spectra of VEGF iM_{WT} at the pka exposed to 0 KCl (mM) / 0% acetonitrile, 100 mM KCl by itself, 40% acetonitrile by itself, and both 100 mM KCl / 40% acetonitrile present. Each spectra was collected at 20 °C, the determined T_M, and 90 °C.

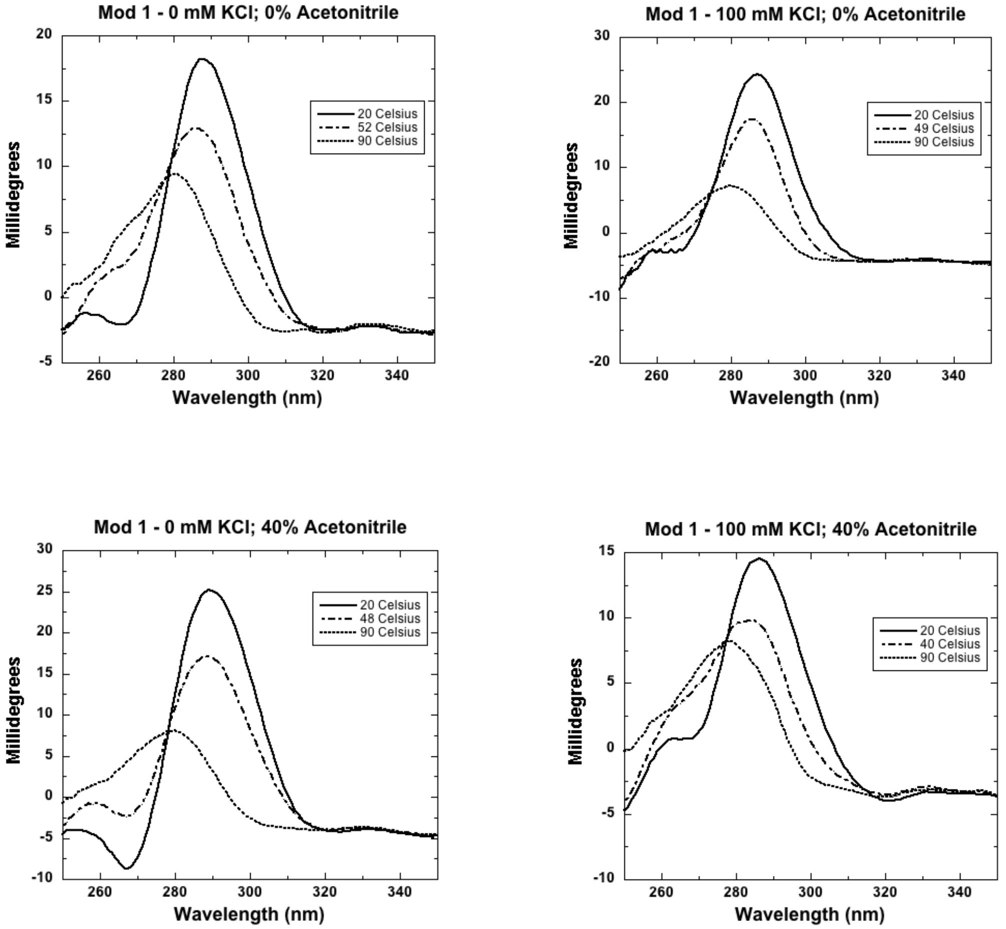

Figure S8. ECD spectra of VEGF iM_{mod1} at the pka exposed to 0 KCl (mM) / 0% acetonitrile, 100 mM KCl by itself, 40% acetonitrile by itself, and both 100 mM KCl / 40% acetonitrile present. Each spectra was collected at 20 °C, the determined T_M, and 90 °C.

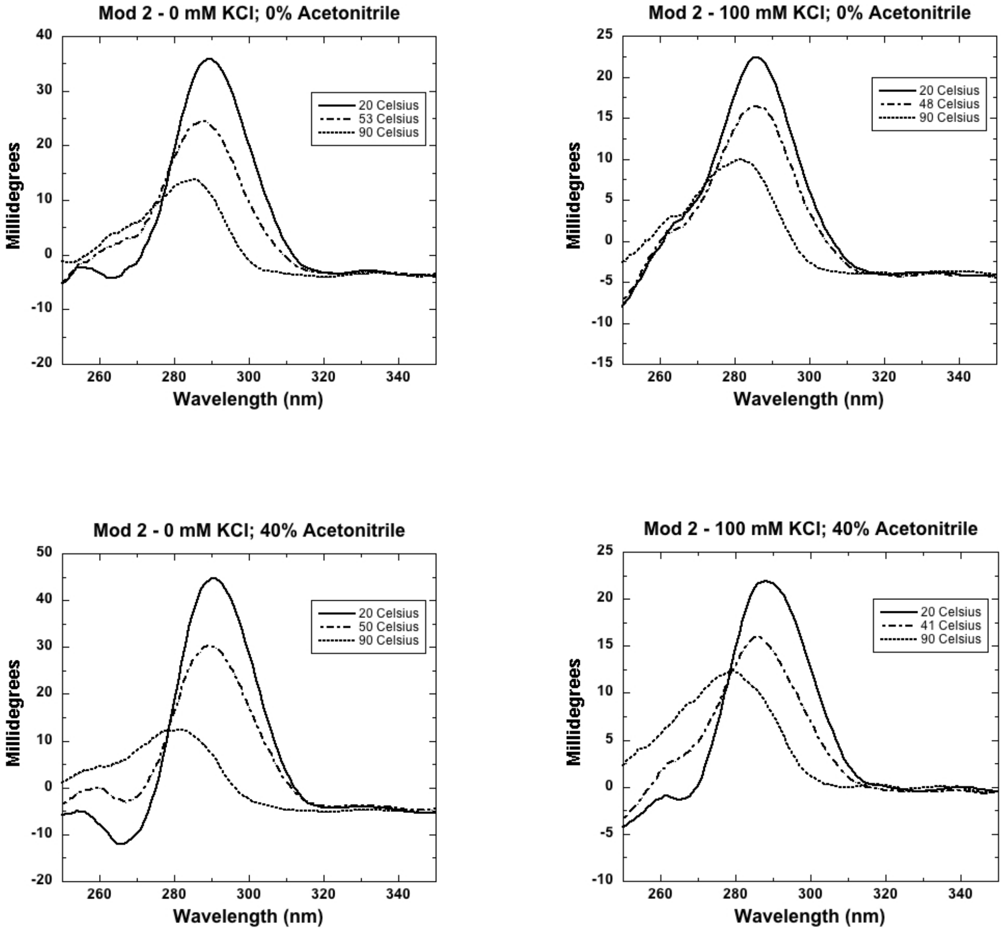

Figure S9. ECD spectra of VEGF iM_{mod2} at the pka exposed to 0 KCl (mM) / 0% acetonitrile, 100 mM KCl by itself, 40% acetonitrile by itself, and both 100 mM KCl / 40% acetonitrile present. Each spectra was collected at 20 °C, the determined T_M, and 90 °C.

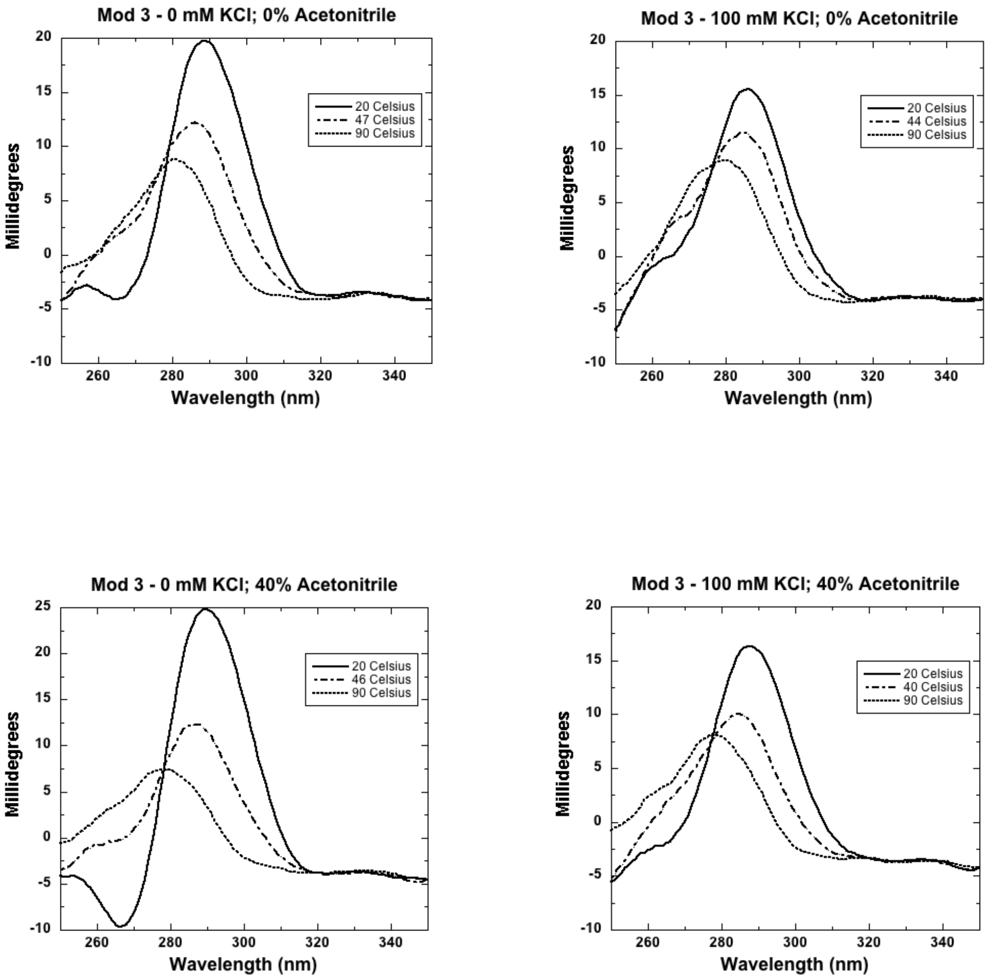

Figure S10. ECD spectra of VEGF iM_{mod3} at the pka exposed to 0 KCl (mM) / 0% acetonitrile, 100 mM KCl by itself, 40% acetonitrile by itself, and both 100 mM KCl / 40% acetonitrile present. Each spectra was collected at 20 °C, the determined T_M, and 90 °C.

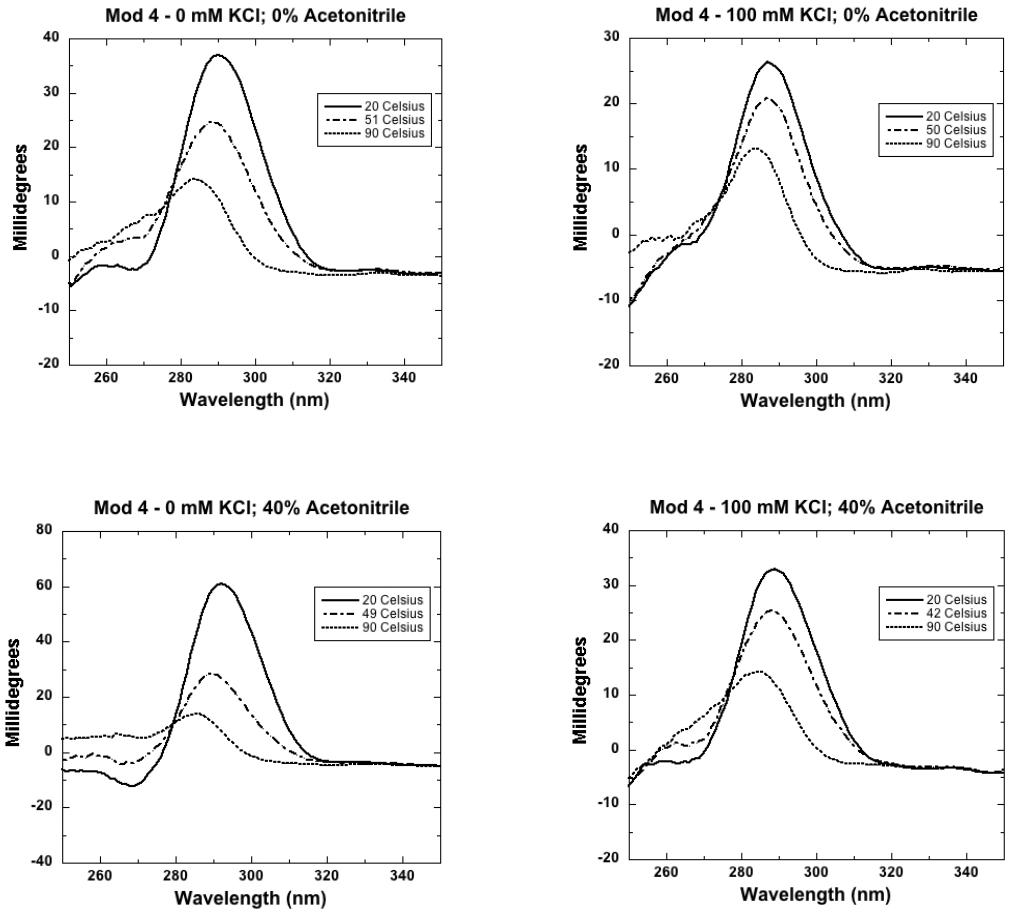

Figure S11. ECD spectra of VEGF iM_{mod4} at the pka exposed to 0 KCl (mM) / 0% acetonitrile, 100 mM KCl by itself, 40% acetonitrile by itself, and both 100 mM KCl / 40% acetonitrile present. Each spectra was collected at 20 °C, the determined T_M, and 90 °C.

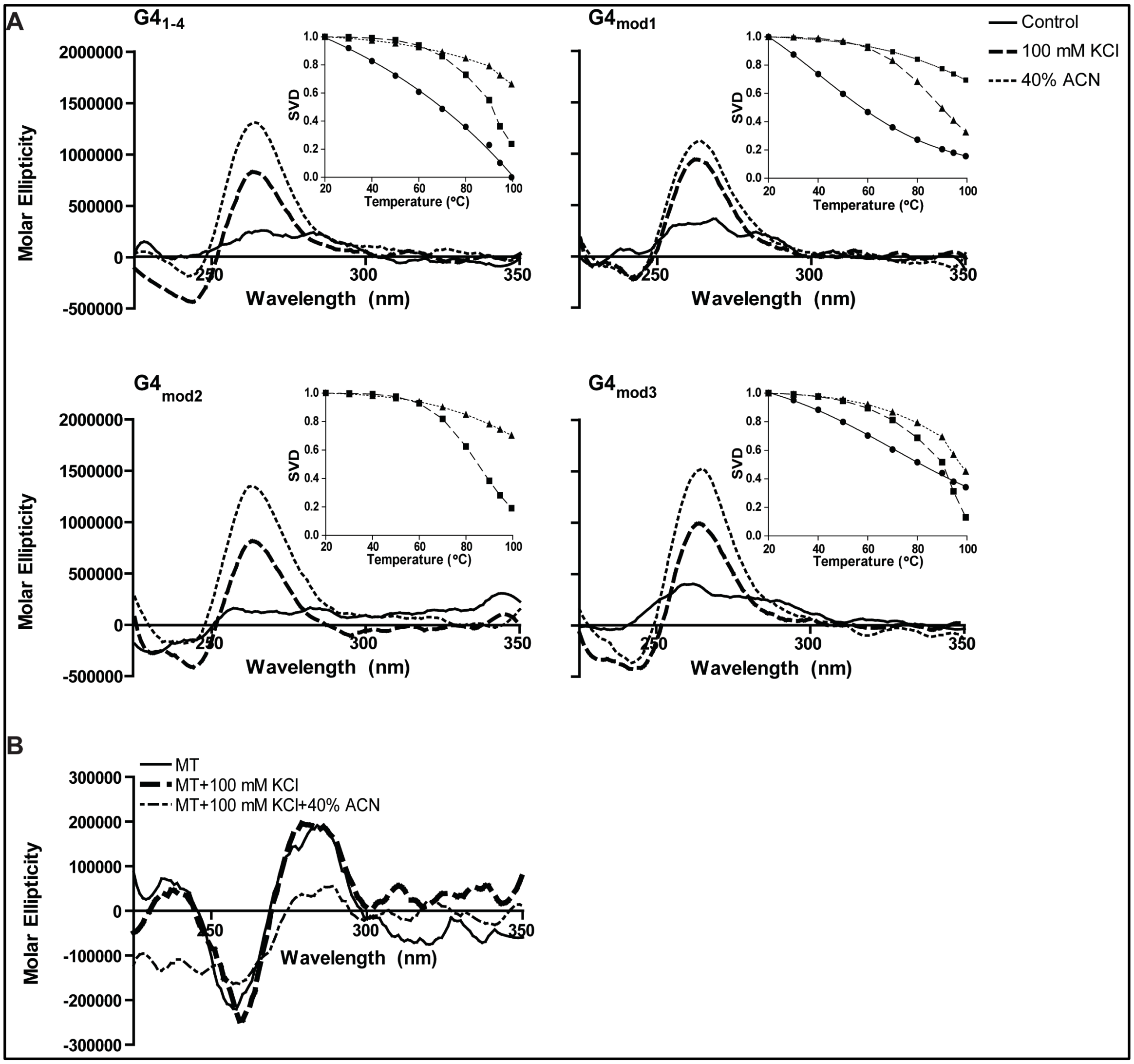

Figure S12. 5-hmC modifications within VEGF G4-forming region allows stable G4 formation with co-solvents. Upon addition of 100 mM KCl alone and combined with 40% ACN, maximal G4 formation occurred compared to control. Also, thermal stability markedly increased with each addition for all sequences (A) Total G-to-T mutations prevent G4 formation alone and in the presence of 100 mM KCl and 40% ACN (B)

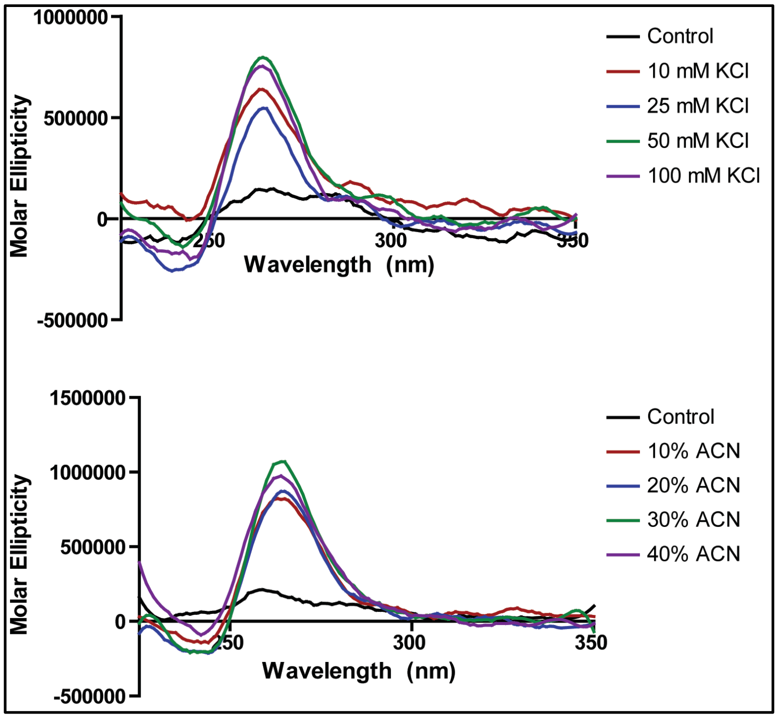

Figure S13. Determination of co-solvent concentrations needed for maximum $VEGF_{1-4}$ formation. 0-100 mM gradient of KCl was added to wild-type VEGF G4 to determine 10 mM KCl to be the minimal concentration affecting G4 formation markedly. Similarly, 0-40% of ACN, in 10% increments, were added to wild-type resulting in 30% ACN being identified for further study.

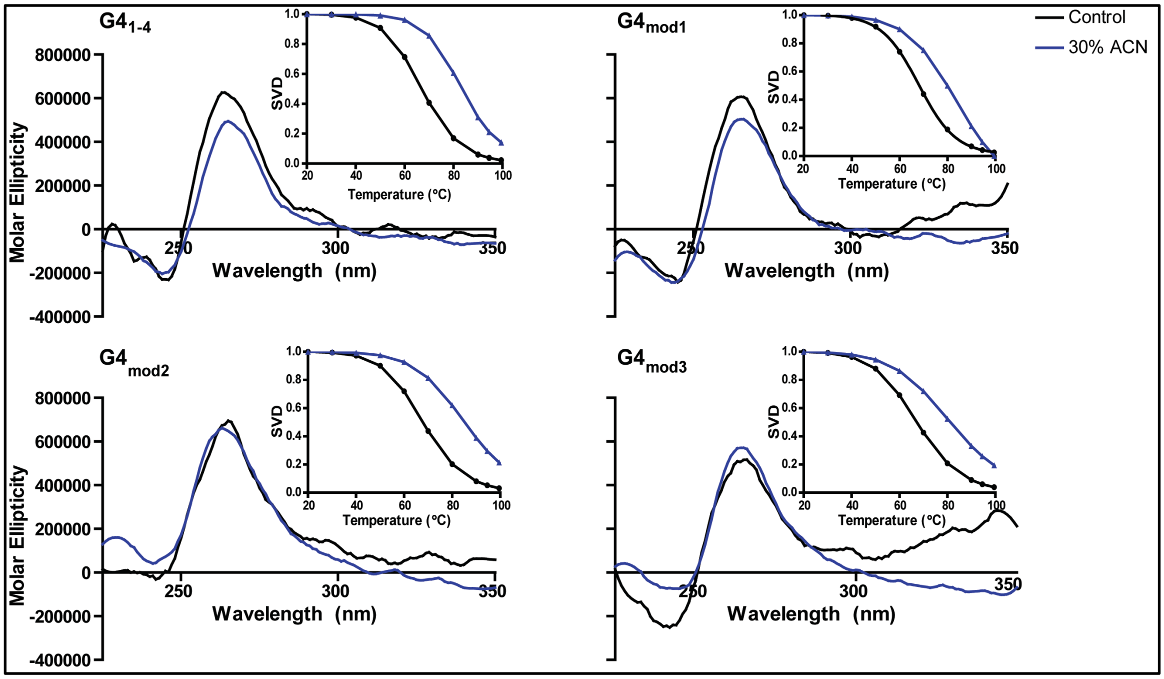

Figure S14. Low concentrations of co-solvents increase thermodynamic stability of modified VEGF structures. 30% ACN increase thermal profiles of each modification as compared to their own control (10 mM KCl)
